# Impact of Obstetric History on Preterm Birth: An Observational Study at a Tertiary Care Hospital in North India

**DOI:** 10.7759/cureus.94558

**Published:** 2025-10-14

**Authors:** Iman Baig, Pavitra K Rastogi, Sujata Deo, Nitu Nigam, Nand Lal, Shilpi Gupta, Nabila Sharif, Rameshwari Singhal, Aisha S Baig, Paridhi Rastogi

**Affiliations:** 1 Department of Periodontology, King George's Medical University, Lucknow, IND; 2 Department of Obstetrics and Gynecology, King George's Medical University, Lucknow, IND; 3 Center for Advanced Research (Cytogenetics Lab), King George's Medical University, Lucknow, IND; 4 Department of Dentistry, Dr. Sone Lal Patel Autonomous State Medical College, Pratapgarh, IND; 5 Department of Conservative Dentistry and Endodontics, Inderprastha Dental College and Hospital, Sahibabad, IND; 6 Department of Conservative Dentistry and Endodontics, Kalka Dental College, Meerut, IND; 7 Department of Dentistry, Babu Banarasi Das College of Dental Sciences, Babu Banarasi Das University, Lucknow, IND

**Keywords:** india, interpregnancy interval, neonatal death, obstetric history, preterm birth, recurrent pregnancy loss, stillbirth

## Abstract

Background

Preterm birth, defined as delivery before 37 weeks of gestation, is a leading cause of neonatal morbidity and mortality worldwide. Obstetric history factors such as prior preterm birth, miscarriages, stillbirths, neonatal deaths, and short interpregnancy intervals have been identified as important predictors, but data from North India remain limited.

Objective

This study aimed to evaluate the association between adverse obstetric history and risk of preterm birth in women delivering at a tertiary care hospital in Lucknow, India.

Methods

This observational study included 200 women aged 18-40 years with singleton live births: 100 preterm (<37 weeks) and 100 term (≥37 weeks). Data on gravidity, parity, abortions, interpregnancy interval, and prior adverse outcomes were collected using a structured proforma. Statistical analysis included chi-squared tests, t-tests, and multivariable logistic regression.

Results

Mean gestational age and birth weight were significantly lower in the preterm group (32.6±2.1 weeks; 1840±510 g) compared with the term group (38.2±1.1 weeks; 2630±520 g; p<0.001). Independent predictors of preterm birth included prior preterm birth (adjusted odds ratio (aOR): 6.41; 95% CI: 2.61-15.72), neonatal death (aOR: 4.66), stillbirth (aOR: 3.87), recurrent abortions (aOR: 3.08), short interpregnancy interval <18 months (aOR: 3.04), and higher gravidity ≥3 (aOR: 2.19). The risk increased cumulatively with multiple adverse factors (aOR: 12.98 for ≥2 risk factors). Model performance was strong (Hosmer-Lemeshow: p=0.57; area under the receiver operating characteristic curve (AUC)=0.80).

Conclusion

Adverse obstetric history is a significant predictor of preterm birth. Systematic documentation of prior obstetric events and targeted antenatal surveillance can help identify high-risk pregnancies and reduce preterm birth burden in resource-limited settings.

## Introduction

Preterm birth, defined as delivery before 37 weeks of gestation, remains a significant public health challenge worldwide, contributing substantially to neonatal morbidity and mortality [1]. Globally, an estimated 15 million babies are born preterm each year, with the highest burden observed in low- and middle-income countries (LMICs) such as India [2]. In India, preterm birth is a leading cause of neonatal deaths, accounting for nearly 35% of the under-five mortality rate [3]. Despite advancements in maternal and neonatal care, the prevalence of preterm birth continues to rise, underscoring the need for targeted interventions to address its underlying risk factors [4].

Obstetric history, encompassing factors such as parity, previous preterm births, miscarriages, stillbirths, and interpregnancy intervals, has been widely recognized as a critical determinant of pregnancy outcomes [5]. Studies have consistently demonstrated that women with a history of preterm birth are at a significantly higher risk of experiencing subsequent preterm deliveries [6]. Past abortions, whether spontaneous or induced, may compromise uterine or cervical integrity and predispose to subsequent adverse outcomes. Recurrent pregnancy loss (RPL) or miscarriages often reflect underlying anatomical, hormonal, or immunological vulnerabilities that can hinder the continuation of future pregnancies. High parity is associated with maternal depletion, cumulative reproductive strain, and cervical changes, all of which increase the likelihood of preterm birth. Short interpregnancy intervals reduce the time available for maternal physiological recovery, limiting nutrient reserves and increasing the risk of growth restriction and early delivery [7,8].

In the context of Lucknow, a city in Northern India with a high burden of maternal and neonatal health challenges, there is limited localized data on the association between obstetric history and preterm birth. Existing studies in similar settings have highlighted the role of socioeconomic factors, inadequate antenatal care, and maternal comorbidities in contributing to preterm birth [9,10]. However, the specific impact of obstetric history remains underexplored, particularly in resource-constrained settings where access to comprehensive maternal care is often limited. This study aims to address this knowledge gap by examining the impact of obstetric history on preterm birth among women delivering at a tertiary care hospital in Lucknow. By comparing the obstetric histories of 100 women with preterm deliveries to those of 100 women with term births, the study seeks to identify specific obstetric factors that may increase the risk of preterm birth. The findings of this study will contribute to a deeper understanding of the predictors of preterm birth in this population and inform targeted interventions to improve maternal and neonatal health outcomes. The present article represents a secondary analysis of data originally collected under a parent study on the relationship between periodontitis, preterm birth, and Fcγ receptor polymorphisms. Since detailed obstetric histories were comprehensively obtained in that study, the current work leverages the same dataset to address an independent but related research question: the role of obstetric history in preterm birth.

## Materials and methods

Study design and setting

This observational, clinical study was conducted between June 2024 and February 2025 at Queen Mary's Hospital, King George's Medical University, Lucknow, India. The study population comprised 200 women: 100 who delivered preterm (<37 completed weeks of gestation) and 100 who delivered at term (≥37 weeks). Women aged 18-40 years with singleton pregnancies resulting in live births were eligible for inclusion. Exclusion criteria were multiple gestations, medically indicated preterm births (e.g., due to pre-eclampsia, eclampsia, placenta previa, placental abruption, or severe intrauterine growth restriction), known fetal anomalies, and pre-existing chronic illnesses.

Sample size

The sample size formula used is as follows: $n=\frac{(Z_{1-&alpha;/2}\sqrt{2P(1-P)}+Z_{1-&beta;}\sqrt{P_{1}(1-P_{1})+P_{2}(1-P_{2})})^{2}}{(P_{1}-P_{2})^{2}}$. Here, $n$ is the required sample size per group, $P_{1}$ is the expected proportion of risk factor in the preterm group, $P_{2}$ is the expected proportion of risk factor in the term group, $P=\frac{P_{1}+P_{2}}{2}$, $Z_{1-&alpha;/2}$ is the Z value for desired confidence level (1.64 for 90% CI), and $Z_{1-&beta;}$ is the Z value for power (0.84 for 80% power).

The sample size formula used was adapted from equation 8.27 of Bernard's Research Methods in the Social Sciences, Fifth Edition [11]. Based on prior estimates of obstetric risk factors for preterm birth in Indian and international studies [9,10], a minimum sample size of 182 women was calculated. After adjusting for a 10% attrition rate (n=18), the final sample size was set at 200 women (100 in the preterm group and 100 in the term group).

The present work is a secondary analysis based on data collected under a parent study that recruited 200 women (100 preterm and 100 term deliveries) according to predefined inclusion and exclusion criteria. The sample size of 200 was originally calculated for the parent study with an expected difference of key risk-factor proportions between preterm and term groups yielding an 80% power at a 90% confidence level. Although the current analysis addresses a different research question, assessing the role of obstetric history, the same sample provides sufficient statistical power for detecting moderate-to-strong associations (odds ratio (OR): ≥2.0) observed in our results. Therefore, no additional sample size recalculation was required.

Data collection

Sociodemographic, medical, and obstetric information was collected at admission using a structured, interviewer-administered proforma (Table 1). Variables of interest included maternal age, gravidity, parity, history of abortion, interpregnancy interval, and prior adverse obstetric outcomes (preterm birth, stillbirth, neonatal death, and RPL). Gestational age was determined from the last reliable menstrual period (LMP), corroborated by early ultrasonography. Birth weight was measured immediately after delivery using a calibrated digital scale.

**Table 1:** Structured questionnaire for women with previous pregnancies

Domain	Question	Response format/details collected
Age at first pregnancy	How old were you when you first became pregnant?	Numeric (years)
Miscarriage history	Have you ever had a miscarriage? If yes, how many?	Yes/no; number
Induced abortion	Have you ever had an induced abortion? If yes, how many?	Yes/no; number
Stillbirth history	Have you ever had a stillbirth? If yes, record (a) date/month/year, (b) gestational age, and (c) birth weight	Yes/no; details
Neonatal death	Have you ever had a baby born alive that died later? If yes, record (a) date of birth, (b) sex, (c) birth weight, and (d) age at death	Yes/no; details
Living children	How many children do you have (apart from this one)?	Numeric
Childbirth details	Give the month and year of birth of each previous child	Details

Definitions followed established criteria as per the World Health Organization (WHO) and American College of Obstetricians and Gynecologists (ACOG) guidelines: Preterm birth was defined as delivery before 37 completed weeks of gestation [1]. Term birth was defined as delivery between 37 weeks and 41+6 weeks. Stillbirth was defined as intrauterine fetal death at ≥20 weeks of gestation [1]. Neonatal death was defined as the death of a liveborn infant within 28 days of delivery. RPL was defined as two or more consecutive pregnancy losses before 20 weeks [1,12]. Short interpregnancy interval was defined as conception occurring within 12 months of a previous live birth [13].

Data collection, analysis, and manuscript preparation were conducted by the dental team, with collaboration and clinical oversight from a co-author in obstetrics and gynecology. No interventions were performed, and no professional boundaries or ethical standards were compromised. This approach ensured the dataset was developed responsibly and interpreted with clinical accuracy.

Statistical analysis

Data were entered into Microsoft Excel (Microsoft, Redmond, WA, USA) and analyzed using IBM SPSS Statistics for Windows, V. 21.0 (IBM Corp., Armonk, NY, USA). Continuous variables were expressed as mean±standard deviation (SD) and compared using the independent t-test. Categorical variables were presented as frequencies and percentages and compared using the chi-squared test or Fisher's exact test where appropriate. Variables showing statistical significance (p<0.05) in univariate analysis were entered into a multivariable logistic regression model to identify independent predictors of preterm birth. Model performance was assessed using calibration (Hosmer-Lemeshow goodness-of-fit test) and discrimination (area under the receiver operating characteristic curve (AUC)). A p-value of <0.05 was considered statistically significant.

Ethical considerations

The study protocol was reviewed and approved by the Institutional Ethics Committee of King George's Medical University (approval number: 16/Ethics/2024). Ethical approval for the parent study encompassed the data collection procedures used here. The parent study, titled "To Explore the Relationship Between Periodontitis and Preterm Delivery With Fcγ Receptor Polymorphisms as Genetic Susceptibility Factor," included a comprehensive collection of obstetric, medical, and sociodemographic data. Written informed consent was obtained from all participants prior to inclusion in the study, and they were assured that all personal and medical information collected would remain strictly confidential and would be used solely for research purposes.

The current article is a secondary observational analysis based solely on these existing, ethically approved data. No additional recruitment, patient contact, or data collection occurred beyond the scope of the approved protocol. Accordingly, no further ethical approval was required.

## Results

Characteristics of the study participants

The demographic and obstetric profile of the study participants revealed key differences between the preterm and term groups (Table 2). Although maternal age did not differ significantly (p=0.20), the mean gestational age and birth weight were significantly lower in the preterm group (32.6±2.1 weeks; 1840±510 g) compared to the term group (38.2±1.1 weeks; 2630±520 g), both with p<0.001. Gravidity, parity, and history of abortions were significantly associated with preterm birth. A higher proportion of multigravidas and parous women was observed in the preterm group, along with a significantly higher history of previous abortions (p=0.011), suggesting that obstetric history plays a critical role in preterm birth risk.

**Table 2:** Characteristics of the study participants Statistical tests: independent t-test for continuous variables and chi-squared test for categorical variables. The asterisks indicate statistical significance at p<0.05.

Characteristic	Total (n=200)	Preterm group (n=100)	Term group (n=100)	P-value (independent t-test, chi-squared test)
Maternal age (years)	25.9±4.1	26.2±4.3	25.6±3.9	0.20
Gestational age (weeks)	35.4±3.3	32.6±2.1	38.2±1.1	<0.001
Birth weight (grams)	2235±670	1840±510	2630±520	<0.001
Gravidity (%)				
Primigravida (G1)	87 (43.5%)	30 (34.5%)	57 (65.5%)	0.004*
Multigravida (G≥2)	113 (56.5%)	70 (61.9%)	43 (38.1%)	
Parity (%)				
Nulliparous	94 (47.0%)	34 (36.2%)	60 (63.8%)	<0.001*
Parous	106 (53.0%)	66 (62.3%)	40 (37.7%)	
Previous abortions (%)				
None	132 (66.0%)	56 (42.4%)	76 (57.6%)	0.011*
≥1 abortion	68 (34.0%)	44 (64.7%)	24 (35.3%)	

Obstetric history factors and preterm birth risk

Several obstetric history factors were strongly associated with an increased risk of preterm birth (Table 3). Prior preterm birth exhibited the highest odds (OR: 6.71; 95% CI: 2.77-16.24), followed by prior neonatal death (OR: 5.24), prior stillbirth (OR: 4.15), and recurrent spontaneous abortions (OR: 3.58). Additionally, short interpregnancy interval (<18 months) and higher parity (≥3 pregnancies) also significantly elevated the risk. These findings underscore the importance of detailed obstetric history in identifying high-risk pregnancies.

**Table 3:** Obstetric history factors and preterm birth risk OR: odds ratio; CI: confidence interval The asterisks indicate statistical significance at p<0.05.

Risk factor	Preterm group (n=100)	Term group (n=100)	OR (95% CI)	P-value
Prior preterm birth	33 (33.0%)	7 (7.0%)	6.71 (2.77-16.24)	<0.001*
≥2 spontaneous abortions	26 (26.0%)	9 (9.0%)	3.58 (1.61-7.94)	0.001*
Prior stillbirth	21 (21.0%)	6 (6.0%)	4.15 (1.60-10.77)	0.003*
Prior cesarean section	38 (38.0%)	25 (25.0%)	1.84 (1.01-3.34)	0.045*
Interpregnancy interval <18 months	43 (43.0%)	19 (19.0%)	3.20 (1.69-6.05)	<0.001*
Prior neonatal death	25 (25.0%)	6 (6.0%)	5.24 (2.00-13.71)	<0.001*
≥3 previous pregnancies	36 (36.0%)	18 (18.0%)	2.57 (1.34-4.91)	0.004*

Multivariable analysis of preterm birth predictors

After adjusting for maternal age and parity, six independent predictors of preterm birth remained significant (Table 4). Prior preterm birth (adjusted odds ratio (aOR): 6.41), history of neonatal death (aOR: 4.66), prior stillbirth (aOR: 3.87), recurrent spontaneous abortions (aOR: 3.08), short interpregnancy interval (aOR: 3.04), and higher gravidity (≥3) (aOR: 2.19) were all significantly associated with increased odds of preterm delivery. The model demonstrated good fit (Hosmer-Lemeshow: p=0.57) and excellent discriminatory ability (AUC=0.80), validating the robustness of the identified predictors.

**Table 4:** Multivariable analysis of preterm birth predictors Model adjusted for maternal age and parity. Hosmer-Lemeshow test: χ²=6.72; p=0.57. Model AUC=0.80 (95% CI: 0.74-0.86). The asterisks indicate statistical significance at p<0.05. aOR: adjusted odds ratio; AUC: area under the receiver operating characteristic curve; CI: confidence interval

Independent predictor	aOR	95% CI	P-value
Prior preterm birth	6.41	2.61-15.72	<0.001*
Interpregnancy interval <18 months	3.04	1.52-6.07	0.002*
≥2 spontaneous abortions	3.08	1.28-7.42	0.012*
Prior stillbirth	3.87	1.37-10.91	0.011*
Prior neonatal death	4.66	1.66-13.10	0.003*
Gravidity ≥3	2.19	1.11-4.33	0.024*

History of pregnancy loss and preterm birth

The history of any pregnancy loss significantly increased the risk of preterm birth (OR: 2.59; p=0.001) (Table 5). RPL (≥2 consecutive losses) was an even stronger predictor (OR: 3.87; p<0.001). The combined history of stillbirth and neonatal death also posed a significant risk (OR: 5.71; p=0.004). These results highlight the critical need to screen and monitor women with a history of pregnancy loss more closely during subsequent pregnancies.

**Table 5:** History of pregnancy loss and preterm birth Recurrent pregnancy loss defined as ≥2 consecutive losses. The asterisks indicate statistical significance at p<0.05.

Pregnancy loss history	Preterm group (n=100)	Term group (n=100)	OR (95% CI)	P-value
Any pregnancy loss	56 (56.0%)	33 (33.0%)	2.59 (1.46-4.58)	0.001*
Recurrent pregnancy loss	32 (32.0%)	11 (11.0%)	3.87 (1.80-8.31)	<0.001*
Stillbirth+neonatal death	15 (15.0%)	3 (3.0%)	5.71 (1.59-20.52)	0.004*

Cumulative risk of obstetric history factors

The risk of preterm birth escalated with the accumulation of adverse obstetric history factors (Table 6). Compared to women with no risk factors, those with one had a threefold increased risk (aOR: 3.05), while those with two or more had a dramatically higher risk (aOR: 12.98). This cumulative effect emphasizes the utility of a risk stratification approach based on prior obstetric history to guide clinical surveillance and interventions.

**Table 6:** Cumulative risk of obstetric history factors *Risk factors: prior preterm birth, ≥2 abortions, prior stillbirth/neonatal death.

Number of risk factors*	Preterm rate	aOR (95% CI)
0	12.0%	Reference
1	29.0%	3.05 (1.38-6.76)
≥2	64.0%	12.98 (5.70-29.52)

## Discussion

In this observational, clinical study from Lucknow, several adverse obstetric history elements, prior spontaneous preterm birth, previous neonatal death, prior stillbirth, RPL, short interpregnancy interval, and higher gravidity, were independently associated with preterm birth, with good model calibration and discrimination (Hosmer-Lemeshow: p=0.57; AUC: 0.80). These findings reinforce the premise that obstetric history is an efficient and feasible risk stratification tool in antenatal care, particularly in resource-limited settings.

Prior preterm birth

Previous preterm birth emerged as the strongest predictor of recurrence (aOR: 6.41), echoing extensive prior evidence. Phillips et al.'s large systematic review and meta-analysis similarly reported markedly increased recurrence risks across diverse populations [14]. The effect size in our cohort is consistent with their results, reinforcing prior preterm birth as a robust, independent risk marker. Our history-based prediction model achieved an AUC of 0.80, which is comparable to AUCs reported for other models that rely solely on historical data [15].

Short interpregnancy interval

We found that a short interpregnancy interval (<18 months) was independently associated with preterm birth. This is biologically plausible through mechanisms such as incomplete maternal nutritional repletion, residual inflammation, and psychosocial stress. Our findings are concordant with the meta-analysis by Conde-Agudelo et al., which reported higher risks of preterm birth and other adverse perinatal outcomes with short intervals [7]. Similarly, Ni et al. confirmed that short spacing increases adverse outcomes even among women of advanced maternal age [15]. The WHO recommendation of a ~24-month interval between a live birth and the next conception provides a policy-relevant framework for counseling [16]. Together, these results underscore the need for reinforcing healthy birth spacing as a preventive measure.

Prior stillbirth and neonatal death

Our study also found previous stillbirth and neonatal death to be strong predictors of subsequent preterm birth. Deng et al. confirmed this association in their systematic review and meta-analysis, demonstrating higher risks of preterm birth and perinatal mortality in pregnancies following a stillbirth [17]. While evidence specifically linking prior neonatal death to preterm birth is less abundant, shared pathways such as placental insufficiency and infection likely contribute. These findings highlight the importance of tailored surveillance and individualized care for women with a history of adverse perinatal outcomes.

RPL

We observed a significant association between RPL and preterm birth (aOR: 3.08), consistent with the systematic review by Wu et al., who reported that RPL increases the risk of preterm birth and other adverse outcomes in subsequent pregnancies [18]. Potential mechanisms include uterine anomalies, cervical insufficiency, endocrine-immune dysfunction, and subclinical infection. This emphasizes the value of comprehensive evaluation and early identification of modifiable contributors in women with RPL.

Parity and gravidity

Higher gravidity (≥3) was associated with preterm birth in our cohort. Literature on parity and preterm birth remains mixed. Koullali et al. reported that both nulliparity and very high parity were linked to increased preterm birth risk, although confounding factors such as maternal age and prior cervical procedures complicate interpretation [19]. Our results suggest that adverse cumulative obstetric history, rather than parity alone, provides a clearer indicator of risk.

Clinical implications

Collectively, these findings support a practical, history-triggered antenatal care strategy: (i) documenting adverse history at booking to flag women at risk; (ii) offering cervical-length (CL) surveillance in mid-trimester for women with prior preterm birth; and (iii) applying evidence-based interventions when indicated. Current guidance from ACOG (Practice Bulletin No. 234, 2021) and its 2023 Practice Advisory emphasizes CL-based management. Progestogens are no longer recommended solely for a history of preterm birth without a short cervix, and 17-alpha hydroxyprogesterone caproate (17-OHPC) is not supported following recent evidence and regulatory withdrawal [12,13]. For women without prior preterm birth but with a CL ≤25 mm, the Society for Maternal-Fetal Medicine (SMFM) recommends vaginal progesterone and selective use of cerclage [20]. Embedding such protocols into antenatal care, while strengthening perinatal audit systems (structured reviews of maternal and neonatal outcomes to identify avoidable factors and improve care), can help operationalize risk reduction pathways in both tertiary and community settings.

Study limitations

This single-center study with a modest sample size may limit generalizability. Reliance on maternal recall introduces potential bias, and unmeasured confounders such as socioeconomic or nutritional factors could have influenced the findings. As an observational study, it establishes association but not causality. As this study represents a secondary analysis of data from an unpublished parent project, potential biases related to the original data collection process and variable definitions cannot be completely excluded. However, the use of predefined inclusion criteria, standardized data collection tools, and rigorous statistical analyses helped minimize such bias and ensure the validity of findings.

## Conclusions

This study shows that a woman's obstetric history, particularly previous preterm birth, neonatal death, stillbirth, RPL, and short interpregnancy interval (<18 months), is strongly linked to the risk of preterm birth in the current pregnancy. Higher gravidity was also an independent predictor. A simple history-based model provided good prediction, highlighting the value of routinely capturing these details during antenatal care.

Emphasizing such history-based screening during antenatal care may assist clinicians in the early identification and closer monitoring of high-risk pregnancies, especially in resource-limited settings. Further large-scale, multi-center studies are recommended to validate these associations and refine predictive models for preterm birth.
